# ZnO thin films made by sputtering: room temperature ferromagnetism due to Zn defects/vacancies?

**DOI:** 10.1039/d5ra00146c

**Published:** 2025-03-21

**Authors:** Nguyen Hoa Hong, Nguyen Sy Pham, Tatsuya Murakami, Mojmir Meduna, Ondrej Caha, Ivana Miháliková, Martin Friák

**Affiliations:** a Department of Condensed Matter Physics, Faculty of Science, Masaryk University Kotlářská 2 Brno 611 37 Czechia hong.nguyen@mail.muni.cz; b Center for Nano Materials and Technology, Japan Advanced Institute of Science and Technology 1-1 Asahidai Nomi Ishikawa 923-1292 Japan; c Institute of Physics of Materials, v. v. i., Czech Academy of Sciences Žižkova 22 Brno 616 00 Czechia

## Abstract

Unlike TiO_2_ and SnO_2_, room temperature ferromagnetism in pristine ZnO films does not appear to originate from oxygen vacancies. In this study, we investigated thin films of ZnO deposited on *R*-cut Al_2_O_3_ by sputtering. The ZnO films were ferromagnetic, with a very high *T*_C_ of about 800 K and were quite magnetically homogenous. Our experiments were complemented by quantum-mechanical calculations of both bulk wurtzite-structure ZnO and its (0001) surfaces, with and without Zn vacancies. While the bulk ground state and the bulk-terminated, vacancy-free (0001) surfaces were non-magnetic, a higher concentration of Zn vacancies deep beneath the surface was shown to contribute magnetic moments to the ferromagnetic state of ZnO.

## Abstract 2

Unlike TiO_2_ and SnO_2_, room temperature ferromagnetism in pristine ZnO films does not appear to originate from oxygen vacancies.

## Graphical abstract

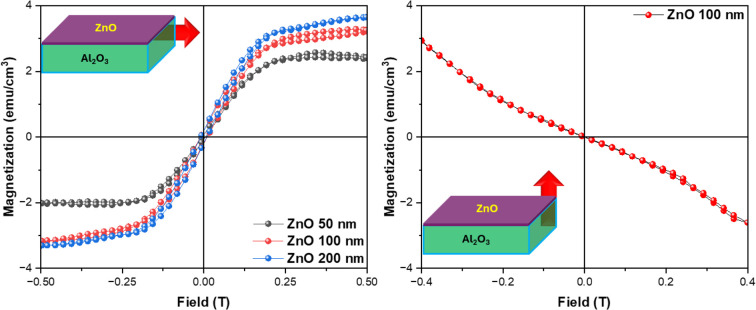

## 1. Introduction

Since the theoretical paper by Dietl *et al.* on the possibility of achieving ferromagnetic semiconductor with high *T*_C_ in ZnO by doping with Mn or other transition metals (TMs) to induce a double-exchange (DE) interaction of 3d electrons that induces magnetism,^1^ many research groups have eagerly explored this direction, hoping to find a new family of materials for spintronics.^2,3^ In 2004, Coey *et al.* reported room temperature ferromagnetism (FM) in undoped HfO_2_, and since then, researchers have become aware of the secondary role of TM doping in inducing FM in semiconducting oxides.^4^ In the following years, several other groups reported this phenomenon of FM in a series of undoped oxides in thin film form, such as TiO_2_,^5,6^ SnO_2_,^7^ In_2_O_3_,^5^ and ZnO.^8,9^ Concerning the role of doping TMs into ZnO, Barla *et al.* reported the paramagnetic contribution of Co doping in ZnO.^10^ Apart from the cases of TiO_2_ and SnO_2_, where oxygen vacancies seem to be the origin of the observed FM,^5,8^ the mechanism in ZnO films appears to be somewhat different. Annealing in an oxygen atmosphere does not have any significant effect on ZnO films made by the pulsed laser deposition (PLD) technique.^8,11^ Experimentally, when modulating the magnetic properties of ZnO films by varying conditions, it was found that a complex defect-assisted mechanism, such as Zn interstitials, might be responsible for the observed room temperature FM in ZnO films without TM doping.^12,13^ Additionally, when using a model based on the tight-binding or linear combination of molecular orbital (LCMO) method to investigate the possibility of FM and high *T*_C_ in semiconducting oxide films due to oxygen vacancies in two-dimensional (2D) configurations, ZnO appears to be governed by a completely different mechanism compared to that of TiO_2_, SnO_2_, or HfO_2_ (ref. 14). It was reported in ref. 8 that FM in ZnO films cannot be attributed to oxygen vacancies but likely to defects on Zn sites, as annealing in an oxygen atmosphere does not change their magnetic properties. Additionally, defects and magnetic moments are reported to be thickness-dependent: for ZnO films made by PLD, magnetization appears to be larger in thin films than in thick films, indicating that if FM is due to vacancies and defects, they must be located mostly at or near the surface and/or interface between the films and substrates.^8,11^ For pure ZnO, the magnetic density of states is negligible unless defects or doping are introduced. The role of defects, such as oxygen vacancies or zinc interstitials, is important in the creation of localized magnetic moments.^13^ Since ZnO is an important semiconductor that has been widely used in next-generation devices, the origin of the observed FM should be clarified, so that it can be manipulated for spintronic applications.^15–17^

In this study, we fabricated ZnO films by sputtering, a method that offers better control compared to the PLD method that we used previously. Simulations were performed to clarify the bulk and surface magnetic states of ZnO. The origin of FM in undoped ZnO thin films will be discussed in detail.

## 2. Experimental section

### 2.1. Sample preparation and measurements

ZnO was deposited on *R*-cut Al_2_O_3_ substrates at room temperature using an ion beam sputtering system (EIS-220, Elionix, Tokyo), applying an Ar pressure of 5 × 10^−3^ PA, microwave power of 1000 W, acceleration voltage of 1000 V, and ion emission current of 0.6 A. The sputtering was performed for 50, 100, and 200 min using a ZnO target, which resulted in 50, 100, and 200 nm thick films, respectively.

Structural studies were done by X-ray diffraction (XRD, Smart Lab 3 kW from Rigaku, Japan) at room temperature. The magnetic moments (*M*) at 300 K *versus* the magnetic field (*H*) from 0 to 0.5 T and *versus* temperature (*T*) at 2 T from 50 K up to 900 K were measured using a vibration sample magnetometry system (VSM, Quantum Design VersaLab). There was an oven option for the VSM system to extend the temperature to 1000 K. The standard working of the VersaLab system (50–400 K) could be extended for the VSM option up to 1000 K due to the addition of the oven kit, for which it was necessary to use a special sample holder. The magnetic field was applied both parallel and perpendicular to the film plane. The chemical compositions of the ZnO films were also analysed by energy-dispersive X-ray (EDX) spectroscopy, since X-ray photoelectron spectroscopy (XPS) was not allowed to be performed to avoid pollution in our ultra-high-vacuum (UHV) cluster. The morphology and chemical analysis were studied by scanning electron microscopy (SEM, TESCAN LYRA 3) and energy-dispersive spectroscopy (EDS, Bruker XFlash 5010), respectively. SEM pictures were taken at a 15 kV accelerating voltage. More detailed investigations were done for assessing the structure of the films with different thicknesses by X-ray reflectivity (XRR) and X-ray absorption spectroscopy (XAS) at room temperature at beamline PM3 of the Synchrotron Center BESSY II. The drain signal was measured at an angle incidence of 4.5°.

### 2.2. Theoretical methodology

Our quantum-mechanical (also called first-principles or *ab initio*) calculations employed the density functional theory (DFT) implemented in the Vienna *Ab initio* Simulation Package (VASP)^18,19^ with the Projector-Augmented-Wave (PAW) pseudopotentials (12-electron Zn and 6-electron O potentials from the VASP database)^20^ and the Generalized Gradient Approximation (GGA) for the exchange–correlation energy. Inspired by the previous results presented in the Materials Project^21,22^ database (item no. mp-2133), the plane-wave energy cut-off was set to 520 eV. Other computational details are given in the Appendix.

## 3. Results and discussion

**Fig. 1:**
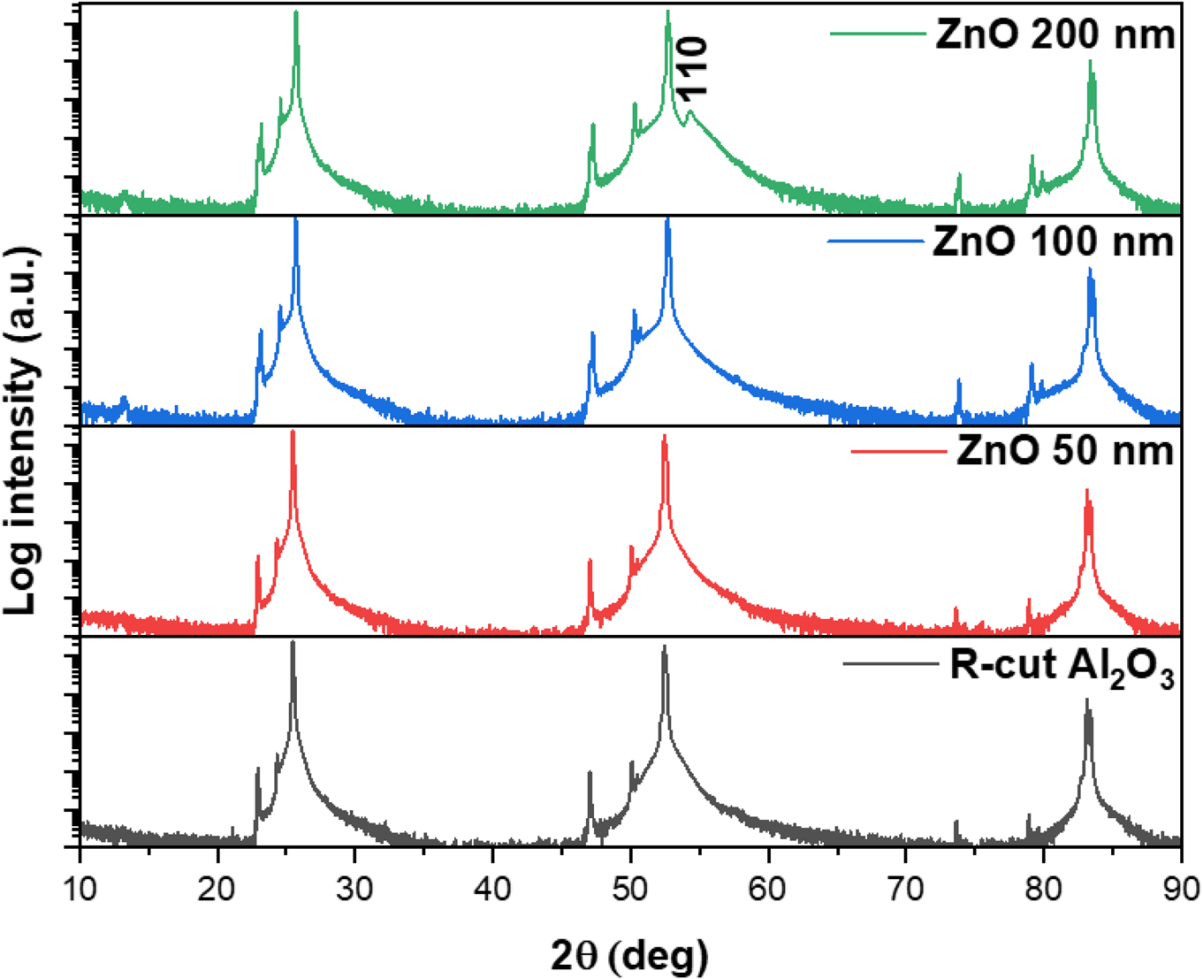
XRD patterns of ZnO films on *R*-cut Al_2_O_3_ substrates with different thicknesses and of a bare *R*-cut-Al_2_O_3_ substrate.

**Fig. 2:**
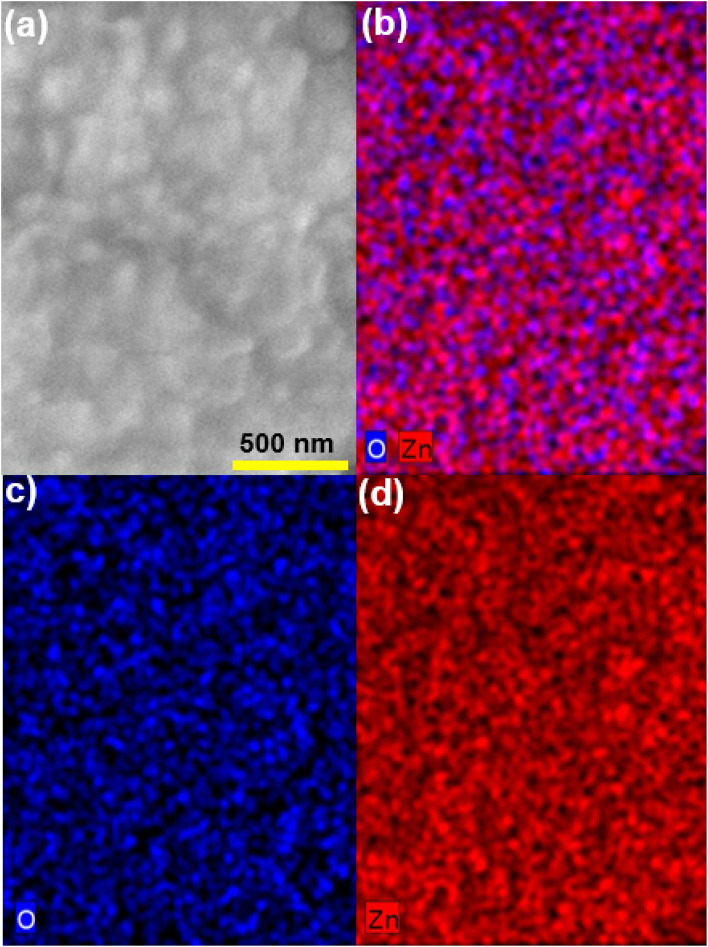
a) SEM image of the 100 nm-thick ZnO film; (b) general color mapping result of the 100 nm-thick ZnO film; (c) Zn color mapping; (d) O color mapping.

**Fig. 3:**
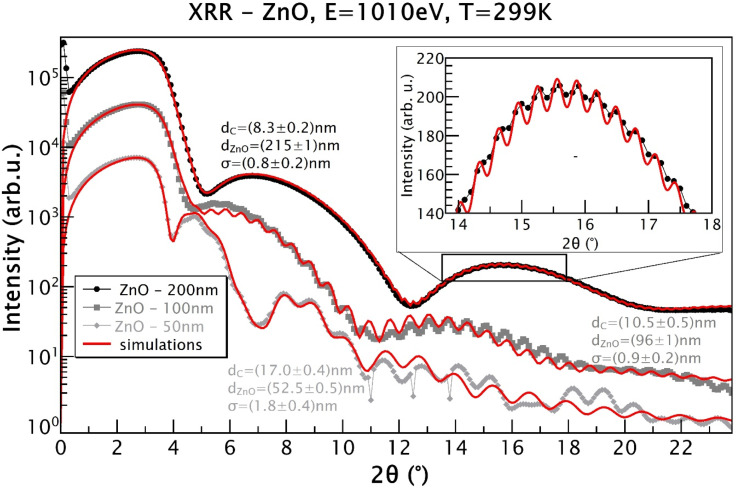
XRR data and simulations for ZnO films with nominal thicknesses of 50, 100, and 200 nm coated by C. *d*_ZnO_ denotes the thickness of the ZnO layer; *d*_C_ represents the C top layer thickness; *σ* represents the highest roughness value of the interfaces obtained from the simulations. The top-right inset shows the detailed zoomed-in image of the XRR curve for the 200 nm-thick ZnO film, showing small and rapid oscillations from the layer. The measurements were realized close to the Zn absorption edge (1021.8 eV) at the X-ray energy of *E* = 1010 eV at 299 K.

All the sputtered ZnO films were colorless and transparent. All the ZnO films with different thicknesses were well crystalized in the wurtzite structure, as displayed in Fig. 1, which shows the X-ray diffraction patterns of the ZnO films as well as a typical *R*-cut Al_2_O_3_ substrate. The morphology of the 100 nm-thick ZnO film is displayed in Fig. 2, indicating its good homogeneity. Additionally, from the energy dispersive spectroscopy (EDS) mapping results shown in Fig. 2(b)–(d) of the ZnO film, great uniform distributions of O and Zn elements can be observed. More sophisticated investigations concerning the structures of the ZnO films were conducted by measuring XRR. Films of different thicknesses were coated with a few nanometers of carbon to enable the XAS measurements, where *d*_ZnO_ denotes the thickness of the ZnO layer and *d*_C_ represents the carbon layer thickness on top, as obtained from the simulations using GenX software.^23^ Additionally, *σ* represents the highest roughness value of the interfaces in the model, as determined from the simulations. The measurements were carried out at 280 K, close to the Sn absorption edge (*e.g.* 484.9 eV) with the X-ray energy set to 480 eV. It can be seen from Fig. 3 that the films grown as 50 nm, 100 nm, and 200 nm films have XRR-simulated thicknesses slightly different from the objective values (note that there was some influence from the C coating here). The top-right inset shows a detailed zoomed-in image of the XRR curve for the 200 nm-thick ZnO sample, showing small and rapid oscillations from the layer. The small rapid oscillations could be explained by the layer being quite thick, whereby the oscillations were very rapid and small in comparison to those of the 100 nm and 50 nm samples. The long-period oscillations (modulation) always came from the top carbon layer, which was much thinner. The measurements were realized close to the Zn absorption edge (1021.8 eV) at an X-ray energy of *E* = 1010 eV and a temperature of *T* = 299 K. The ZnO films were basically quite smooth. The roughness was determined to be (0.8 ± 0.2) nm for the 200 nm-thick film and (0.9 ± 0.2) nm for the 100 nm-thick film, for example.

**Fig. 4:**
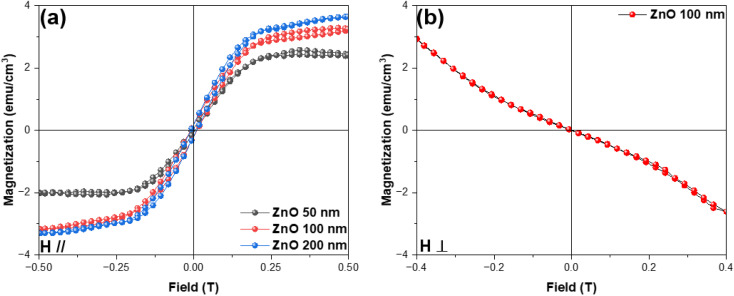
Magnetization *versus* magnetic field at 300 K for (a) ZnO films with different thicknesses when a magnetic field was applied parallel to the film plane; and the (b) 100 nm-thick ZnO film when the magnetic field was applied perpendicular to the film plane.

The magnetization *versus* magnetic field curves obtained at room temperature for the ZnO films, with the field applied parallel to the film plane, are shown in Fig. 4(a). It can be seen that all the films are ferromagnetic at 300 K (it can also be observed that the magnetic moment increases as the field increases, and at some point (*H*_C_), it becomes saturated). The magnitude of the saturated magnetization was in the same order as that of the ZnO films fabricated by the PLD technique reported in ref. 8 by Hong *et al.* (*i.e.*, quite modest, where the maximum saturated magnetization is about 3.8 emu cm^−3^, which is 1 or 2 orders of magnitude less compared to that of other oxides, such as TiO_2_ or SnO_2_, exactly as predicted by the authors in ref. 14). This confirms that room-temperature FM in ZnO is universal and reproducible. One should note that there was a negligible thickness dependence of the magnetization in sputtered ZnO films. For the 50 nm, 100 nm, and 200 nm films, the magnetic moments were 3.13 × 10^−6^ emu; 7.7 × 10^−6^ emu, and 1.76 × 10^−5^ emu, respectively, revealing a linear evolution of the magnetic moment with respect to thickness. This is in contrast to other oxide films, such as TiO_2_ and SnO_2_, whose magnetic properties are surfaced-related, meaning their magnetic moments are similar for different thicknesses. After normalization, the magnetization appeared to much larger for thinner films. That is to say, if the observed FM in undoped ZnO films originates from defects or vacancies, then those defects are not only located at the surface and sub-surface layers, as reported for TiO_2_ (ref. 24) and SnO_2_,^25^ but must also exist in the deeper layers of the films. The *M*–*H* curve at room temperature, measured in a perpendicular configuration for a typical ZnO film (here, a film 100 nm-thick film), is shown in Fig. 4(b). It can be seen that in the perpendicular configuration, the ZnO film is diamagnetic and shows significant magnetic anisotropy. In other words, this somewhat reinforces the assumption that the origin of the observed FM in ZnO may stem from defects/vacancies. Defects and vacancies are normally not distributed evenly in all directions, depending on the crystallization of the films and the texture of each layer.^24–26^ It has been previously reported that, for example, O vacancies or Ti/Sn vacancies must be in a specific plane to contribute to the total magnetic moment.^24,25^ A similar feature has been observed in other undoped oxides. In the case of many undoped semiconducting oxides, there is always a strong anisotropy, whereby strong FM is found in only one direction of the applied magnetic field.^5,26,27^

**Fig. 5:**
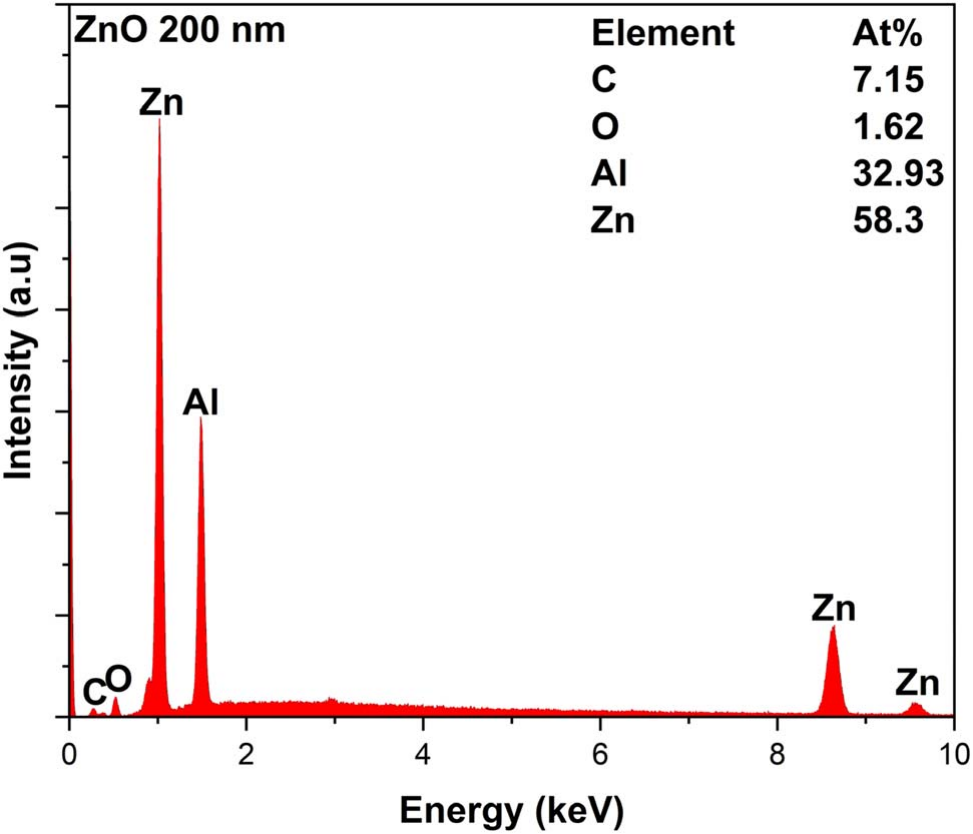
EDX profile for the 100 nm-thick film.

**Fig. 6:**
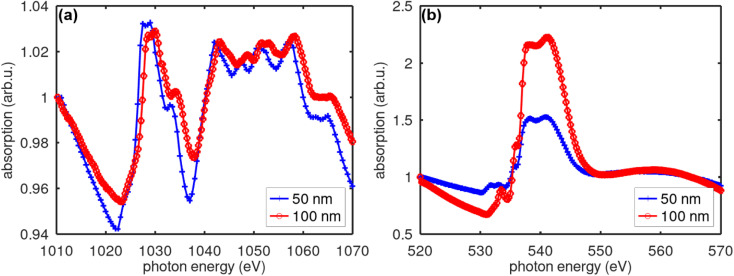
(a) Total electron yield measurement at the Zn-L2,3 absorption edge for two ZnO films with thicknesses of 50 nm and 100 nm at 83 K; and (b) similar measurement at the O–K absorption edge. The measurement was performed at an angle of incidence of 4.5°. The film was coated by a 10 nm-thick carbon layer to prevent sample charging during the measurement.

The EDX spectrum of the 200 nm-thick ZnO film (which has the largest *M*_s_) is shown in Fig. 5. It can be seen that the O : Zn ratio detected is 1.62 : 58.6, while it should be 1 : 1, objectively. The C peak was related to the sample holder. Note that an Al peak existed in the spectrum, indicating that the result also included the substrate composition. When oxygen was present in the substrate as well, it could be seen that the oxygen amount detected from the film must be smaller than 1.62. Since the EDX does not allow us to discuss quantities precisely, we can only say that there are some significant deviations from stoichiometry for both O and Zn. The XAS data are shown in Fig. 6. Fig. 6(a) shows the total electron yield measurement performed at the Zn-L2,3 absorption edge of two ZnO films with different thicknesses at 83 K, while Fig. 6(b) shows a similar measurement at the O–K absorption edge. The XAS spectra at the Zn edge of the two films with different thicknesses did not show any obvious difference, indicating a uniform distribution of Zn along the thickness, while this was not the case when observed at the O–K edge. The Zn-L2,3 edge showed splitting into several peaks corresponding to the oxidation states of zinc. The Zn 2p_3/2_ core level appeared at about 1029 eV, which was similar to what ref. 28 reported. The situation for the O edge was quite complicated, as there was oxygen in both the film and the substrate. When compared with the XAS data for Zn metal reported in ref. 29, we found that the XAS data of our ZnO films did not show a spectrum located at the position of the Zn metal spectrum or corresponding to the Zn^2+^ oxidation state. Therefore, we could conclude that our films were free from Zn metal clusters, and thus, the observed FM could not be attributed to Zn clusters.

**Fig. 7:**
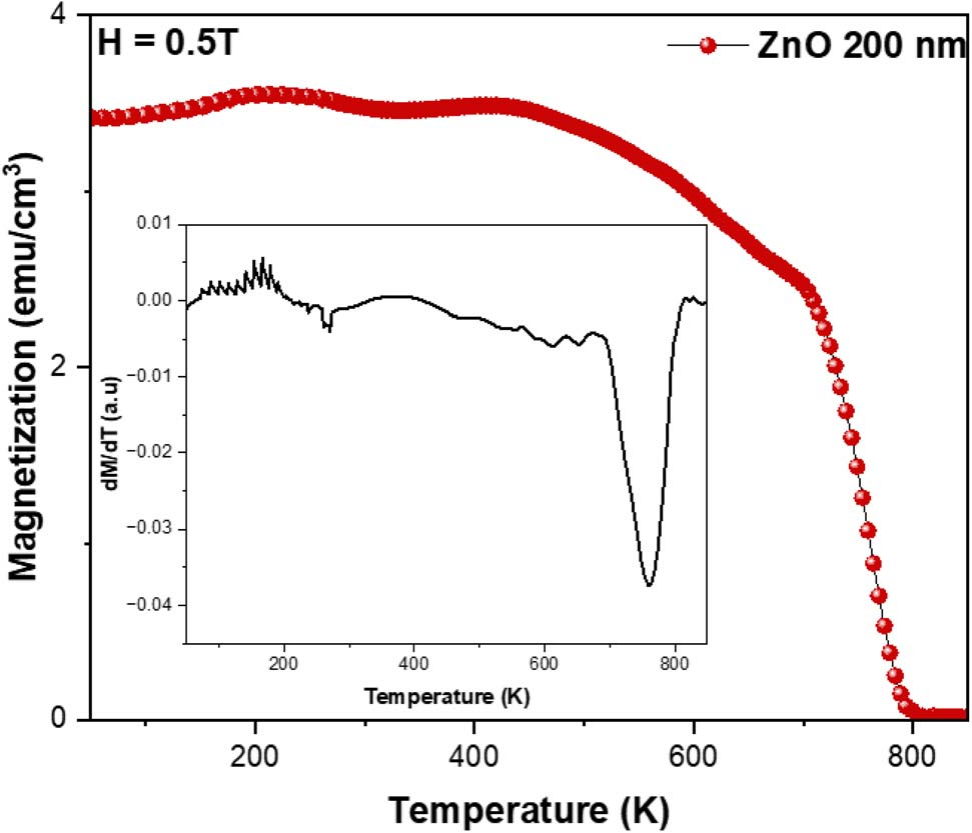
Magnetization *versus* temperature at 0.5 T for the 100 nm-thick ZnO film when a magnetic field was applied parallel to the film plane. The insert shows d*M*/d*T versus T*.

Theoretically, assuming that DE interactions in Mn-doped ZnO could induce FM in ZnO, Dietl *et al.* predicted that the Curie temperature (*T*_C_) in Mn–ZnO could be above room temperature (approximately 300 K).^1^ However, there has been no reported observation of *T*_C_ for undoped ZnO so far, possibly due to the limitations of current magnetometers, which generally have not been equipped with high-temperature ovens. We report herein the magnetization *versus* temperature data taken at 0.5 T for the 200 nm-thick ZnO film within the temperature range from 50 to 900 K. It can be seen from Fig. 7 that the ZnO film has a *T*_C_ of about 800 K. The inset shows the plot of d*M*/d*T versus T*, confirming that the phase transition from paramagnetic to ferromagnetic starts at around 760 K. One must note here that there is no Mn or other 3d doping in undoped ZnO films; thus, the magnetic interaction responsible for such a high *T*_C_ cannot be DE interaction but must have a completely different nature.

**Fig. 8:**
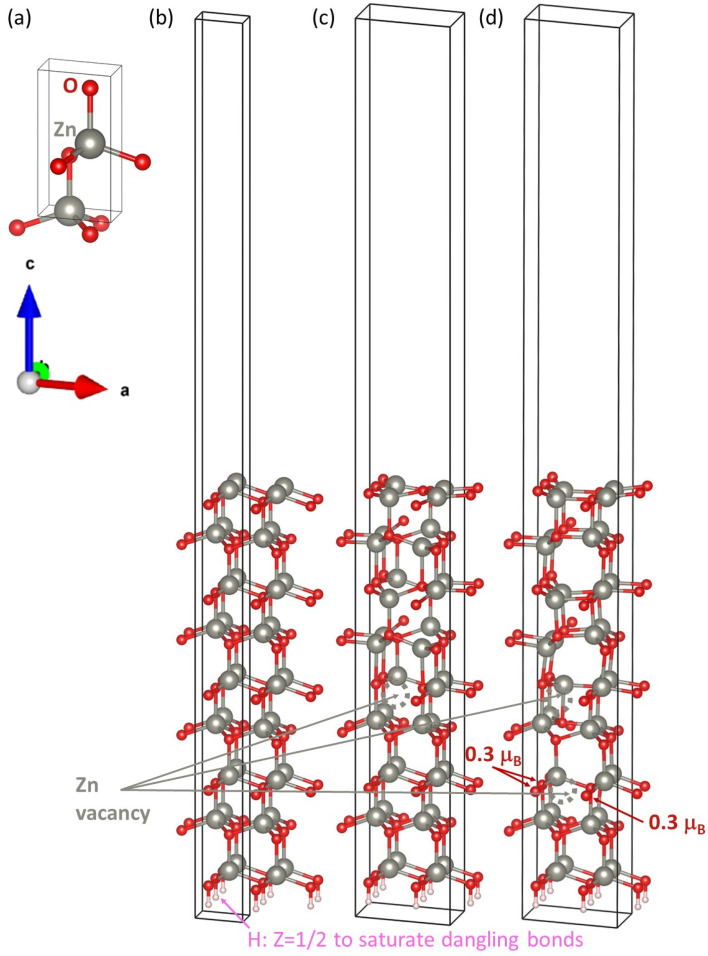
Schematic of the computational cells: bulk ZnO with the hexagonal wurtzite structure (a), vacuum-containing (0001)-oriented surface (1 × 1) slab cell without Zn vacancies (b) and surface (2 × 2) slab cells containing one Zn vacancy (c) or two Zn vacancies (d). The surface slab cells have their lower surfaces covered by half-electron H atoms to saturate the dangling bonds. Zinc vacancies are indicated by grey hashed circles. There are more atoms shown in part (b) because it is presented as a quadruple of the actual surface (1 × 1) cell for a direct comparison with parts (c and d). Part (d) also contains the values of the local magnetic moments localized in 3 oxygen atoms close to the lower Zn vacancy.

Next, to clarify the atomic origin of the magnetic states in ZnO, we performed a series of *ab initio* calculations. We started with the ZnO bulk with the wurtzite structure (space group *P*6_3_/*mc*) by computing its ground-state properties. The 4-atom unit cell used is shown in Fig. 8(a). The calculated equilibrium lattice parameters *a* = *b* = 3.281 Å and *c* = 5.298 Å were in good agreement with our X-ray experimental data (*a* = *b* = 3.25 Å and *c* = 5.207 Å). The bulk ZnO was predicted by our calculations to be non-magnetic, although the calculations were initialized as ferromagnetic. This was in good agreement with the experimental findings regarding the diamagnetic behavior of bulk ZnO.^8^

After computing the properties of the ZnO bulk, we proceeded with the calculations for the surfaces. First, regarding the (0001) surfaces, our computational surface (1 × 1) slab cell without vacancies is shown in Fig. 8(b). Here, the lateral dimensions within the (0001) plane were kept equal to the bulk values, *i.e.*, the lattice parameters were *a* = *b* = 3.281 Å. Also, note that there are atoms from four of these computational surface (1 × 1) slab cells shown in Fig. 8(b), allowing for a direct comparison with slabs containing vacancies, as described below. In the perpendicular direction of the surfaces, the computational slab shown in Fig. 8(b) contained 9 multiples of the wurtzite-structured bulk ZnO formula unit, *i.e.*, 18 atoms, plus a half-electron H atom at the lower surface to saturate the dangling bonds. These atomic layers spanned about 24 Å, and the slab cell further included about 26 Å of vacuum, resulting in the *c* cell parameter perpendicular to the (0001) surface being equal to 50 Å. The surface slabs visualized in Fig. 8(c) and (d) are (2 × 2) multiples of the surface unit cell with either one Zn vacancy (see Fig. 8(c)) or two Zn vacancies located deep beneath the surface of our computational surface slabs, *i.e.*, inside the ZnO film.

Regarding the bulk-like terminated (0001) surface in Fig. 8(b), it turned out to be non-magnetic, the same as the ZnO bulk. Computations for O vacancies at and below the surface of ZnO indeed did not result in any magnetic moment (results are not shown in the this article). To examine the impact of Zn vacancies inside the ZnO films, we next performed calculations with Zn vacancies deep beneath the studied surface. A single Zn vacancy in a quadrupled (2 × 2) slab is shown in Fig. 8(c), which corresponds to the removal of 25% of the Zn atoms from one of the Zn (0001) atomic planes, leading to a non-magnetic state again. In contrast, a pair of Zn vacancies, as shown in Fig. 8(d), could result in a ferromagnetic state characterized by local magnetic moments of 0.3 *μ*_B_ localized in three O atoms close to the lower Zn vacancy. It is worth noting that the studied system with Zn vacancies was characterized by strongly deformed atomic configurations (see the differences between the structures in Fig. 8(b) and those in Fig. 8(c) and (d)). Consequently, the two-Zn-vacancy states in Fig. 8(c) and (d) are characterized by rather high energies. Considering the fact that the slabs in Fig. 8(c) and (d) contained four times more atoms than the vacancy-free slab in Fig. 8(b) minus the number of missing Zn atoms, it is possible to evaluate the energy increase due to the Zn vacancies with respect to the vacancy-free slab in Fig. 8(b). The difference in the number of Zn atoms was addressed by considering the chemical potential of Zn, which we equated to the energy of one Zn atom in the bulk Zn with the hexagonal close-packed structure. The single Zn vacancy in Fig. 8(c) increased the energy of the slab by 2.15 eV. Importantly, the pair of vacancies in Fig. 8(d), which was associated with the magnetic state, resulted in an energy increase of 6.28 eV with respect to the vacancy-free state. Obviously, the magnetic state had a significantly higher energy per vacancy than the non-magnetic state. The results of the contributions of the magnetic moments from the Zn vacancies deep inside the studied surface seemed to well explain the fact that the FM in ZnO films was certainly not surface-related and well confirmed the comments we made earlier regarding the deformation of the O : Zn ratio when we discussed our experimental data. In ref. 8, regarding ZnO films made by PLD, the observed FM was found not to originate from oxygen vacancies but rather from defects on Zn sites. In our films made by sputtering, we observed a similarity in our findings. However, the magnetic moment that arose from the Zn defects was in a high-energy state, suggesting that it would not be stable. This point needs to be addressed before using the ZnO films for spintronic devices. It would be desirable to compute larger computational cells with more atoms, which may allow for the simulation of lower concentrations of Zn vacancies. For example, the computational cells in Fig. 8(b)–(d) were (2 × 2) multiples of the surface unit cell, and it would be desirable to compute (3 × 3), (4 × 4) or even higher multiples. However, these calculations would be too computationally demanding (*i.e.*, they scale as the square of the number of electrons in atoms when downscaling the number of *k*-points in the reciprocal space), and would be beyond our computational means. We thus leave this topic for our future studies.

## 4. Conclusion

Our sputtered films of ZnO showed well-pronounced ferromagnetism with a very high *T*_C_. There was almost no thickness dependence of the magnetic moment, indicating magnetic homogeneity of the films. The experimental data showed that Zn defects existed in our ZnO films. Our quantum-mechanical calculations of both the bulk wurtzite-structured ZnO as well as its (0001) surfaces with and without Zn vacancies showed that a higher concentration of Zn vacancies deep under the surface could contribute magnetic moments to the ferromagnetic state of ZnO. It is worth noting that the magnetic states related to the Zn vacancies corresponded to a high concentration of Zn vacancies and strongly structure-distorted geometries with relatively high energy.

## Data availability

All data are included in the manuscript. Raw data can be obtained from the authors upon reasonable request.

## Author contributions

The role of N. H. Hong includes obtaining the funding, conceptualization the whole project, interpreting data and writing the manuscript. N. S. Pham has been responsible for measuring XRD, VSM, EDX and EDS-mapping for all samples, analyzing the data and visualizing figures. T. Murakami fabricated the films. M. Meduna and O. Caha have been responsible for measuring XRR and XAS and analyzing those data. I. Miháliková and M. Friák have been performing the computational work, visualizing and writing their respective parts. All authors edited and corrected the final manuscript.

## Conflicts of interest

There are no conflicts to declare.

## Appendix

The reciprocal-space Brillouin zone of the 4-atom wurtzite-structured bulk ZnO was sampled by a 16 × 16 × 10 *k*-point mesh. Regarding the slabs used for modeling the (0001) surfaces of ZnO, the reciprocal space corresponding to the (1 × 1) surface slab shown in Fig. 8(b) was sampled with a 16 × 16 × 1 *k*-point mesh while the quadruple computational (2 × 2) surface slab cells in Fig. 8(c) and (d) had their reciprocal spaces sampled with a 8 × 8 × 1 *k*-point mesh. To properly describe both the (i) non-magnetic semiconducting states and (ii) magnetic states, Gaussian smearing was used (parameter ISMEAR = 0) in combination with the smearing parameter SIGMA = 0.05 eV. The total energy was minimized with respect to the internal atomic positions within the slab cells, whose shape were kept constant. Additionally, we applied monopole/dipole and quadrupole corrections to the total energy (the parameter IDIPOL = 3) in combination with the corrections to the potential and forces (parameter LDIPOL on) to suppress the long-range interactions across the vacuum between the periodic images of the slabs. When performing the calculations, we included non-spherical contributions related to the gradient of the density in the PAW spheres (parameter LASPH = TRUE). To model a semi-infinite material under the studied surfaces, the lower surfaces of the surface slabs shown in Fig. 8 (b)–(d) were covered by half-electron H atoms to saturate the dangling bonds, see ref. 30. Periodic boundary conditions were applied to all our calculations.
